# Generation of induced pluripotent stem cell lines from 3 distinct laminopathies bearing heterogeneous mutations in lamin A/C

**DOI:** 10.18632/aging.100277

**Published:** 2011-03-28

**Authors:** Jenny CY Ho, Ting Zhou, Wing-Hon Lai, Yinghua Huang, Yau-Chi Chan, Xingyan Li, Navy LY Wong, Yanhua Li, Ka-Wing Au, Dongsheng Guo, Jianyong Xu, Chung-Wah Siu, Duanqing Pei, Hung-Fat Tse, Miguel Angel Esteban

**Affiliations:** ^1^ Cardiology Division, Department of Medicine, The University of Hong Kong, Queen Mary Hospital, Pokfulam, China; ^2^ Research Center of Heart, Brain, Hormone & Healthy Aging, Faculty of Medicine, The University of Hong Kong, Pokfulam, China; ^3^ Stem Cell and Cancer Biology Group, Key Laboratory of Regenerative Biology, South China Institute for Stem Cell Biology and Regenerative Medicine, Guangzhou Institutes of Biomedicine and Health, Chinese Academy of Sciences, Guangzhou 510530, China

**Keywords:** reprogramming, induced pluripotent stem cells, lamin A/C, dilated cardiomyopathy, atypical Werner syndrome, Hutchinson Gilford progeria

## Abstract

The term laminopathies defines a group of genetic disorders caused by defects in the nuclear envelope, mostly the lamins. Lamins are the main constituents of the nuclear lamina, a filamentous meshwork associated with the inner nuclear membrane that provides mechanical stability and plays important roles in processes such as transcription, DNA replication and chromatin organization. More than 300 mutations in *lamin A/C* have been associated with diverse clinical phenotypes, understanding the molecular basis of these diseases may provide a rationale for treating them. Here we describe the generation of induced pluripotent stem cells (iPSCs) from a patient with inherited dilated cardiomiopathy and 2 patients with distinct accelerated forms of aging, atypical Werner syndrome and Hutchinson Gilford progeria, all of which are caused by mutations in *lamin A/C*. These cell lines were pluripotent and displayed normal nuclear membrane morphology compared to donor fibroblasts. Their differentiated progeny reproduced the disease phenotype, reinforcing the idea that they represent excellent tools for understanding the role of lamin A/C in normal physiology and the clinical diversity associated with these diseases.

## INTRODUCTION

The mammalian cell nucleus is a highly organized and dynamic organelle, not simply a house for the genome [1]. A number of structures are involved in maintaining this high level of organization, among them the nuclear lamina, a thin protein layer that separates the nuclear envelope from the nuclear interior [2]. The lamina is a filamentous meshwork that regulates nuclear structural integrity, the position and size of nuclear pores, and the anchorage of heterochromatin at the nuclear periphery. It is composed mostly of A- and B-type lamins, a group of intermediate filament-like proteins that also reside in the internal nuclear matrix. There are 2 A-type lamin isoforms, lamin A and C, that originate through alternative splicing of the same gene and only differ in the carboxy-terminal domain [3]. Lamin A is longer but undergoes multistep posttranslational modification resulting in cleavage of part of the carboxy-terminal end. Interestingly, cells behave normally without lamin A but elimination of both lamin A and C is lethal [4], suggesting that the 2 proteins exert redundant functions. Perhaps not unexpectedly, the nuclear lamina is a target for many human diseases, which are termed generically laminopathies [5]. Many of these conditions affect lamin A/C but others are caused by mutations in their associated proteins (e.g. emerin). In contrast, few diseases have been identified so far that are caused by mutations in type B-lamins, though this could simply reflect that such mutations are embryonic lethal [6]. Notably, the clinical variability among *lamin A/C*-associated diseases is surprisingly big for a group of conditions affecting the same gene. It ranges from dilated cardiomyopathy, dermatopathy, osteoskeletal dysplasia and lipodistrophy, to more general progeroid syndromes including atypical Werner syndrome and Hutchinson Gilford progeria. The genotype-phenotype correlations are not yet well understood [7] but in all cases there is abnormal blebbing of the nuclear membrane, which is regarded a hallmark of these diseases [8]. Complicating the study of laminopathies, over-expression of mutated lamin A variants is problematic as it can induce cell senescence [9], and the existing animal models do not reproduce the clinical complexity observed in humans [10].

The reversion of somatic cell fate to a naїve pluripotent state by expressing defined sets of transcription factors is a major scientific advance with implications at multiple levels [11]. This revolutionary discovery by Takahashi and Yamanaka [12] has come in a moment in which there is urgent need for novel *in vitro* molecular biology studies that improve our understanding of many human conditions, including the laminopathies. iPSC cell lines retain the ability to differentiate into specific lineages, enabling the study of a given disease in a patient-specific and cell-specific context [13]. Many questions remain yet unanswered as to how the reprogramming happens and whether iPSCs are identical or not to embryonic stem cells (ESCs) [14], but these concerns seem less relevant for the creation of *in vitro* disease models. Indeed, a series of cell lines have already been established that reproduce relevant aspects of the disease phenotype [15-18], opening a new research era full of possibilities. Our study describes an *in vitro* iPSC-based platform for studying the multi-systemic susceptibility to distinct mutations in *lamin A/C* that could be used as well for testing compounds aimed to correct the abnormalities.

## RESULTS

### Generation of iPSCs from 3 *lamin A/C*-associated diseases

**Figure 1.**
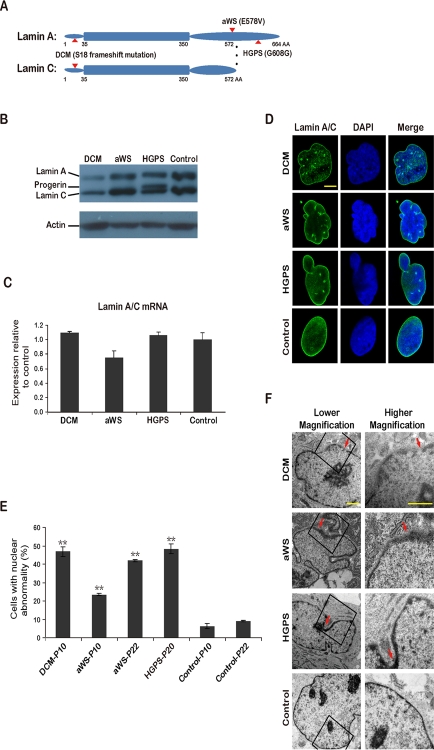
iPSCs from fibroblasts with 3 different mutations in *lamin A/C* **(A)** Simplified scheme depicting lamin A/C proteins and the corresponding amino acid (AA) substitutions (indicated with arrows) in DCM, aWS, and HGPS patients. **(B)** Western blot showing reduced expression of lamin A/C and accumulation of progerin in primary fibroblasts from DCM and HGPS patients respectively. Normal human fibroblasts were used as control. P indicates passage. Actin was used as loading control. A representative experiment is shown (this also applies hereafter when not mentioned otherwise). **(C)** qPCR analysis shows rather similar *lamin A/C* expression in primary fibroblasts from the 3 diseases compared to control fibroblasts. **(D)** Immunofluorescence photographs showing abnormalities of the nuclear membrane in primary fibroblasts from the 3 diseases compared to control fibroblasts. Nuclei are shown in blue. Scale bar indicates 10 μm. **(E)** Quantification of cells displaying abnormal nuclear membrane assessed by immunofluorescence microscopy and cell counting. The mean of 3 independent experiments +/− standard deviation (SD) is shown. ** indicates p value <0.01 measured with Student's t-test. **(F)** Electron microscopy photographs show nuclear membrane abnormalities in more detail. Arrows point to areas with more striking defects. Scale bars indicate 1 μm.

We chose a patient with hereditary dilated cardiomyopathy and conduction system defect, and another one with atypical Werner syndrome, hereafter termed DCM and aWS respectively. To the best of our knowledge these diseases have not been used before for producing iPSCs. Cells from a patient with Hutchinson Gilford progeria, hereafter termed HGPS, were also reprogrammed and used for comparisons. iPSCs from patients with HGPS were reported independently by Zhang et al. [19] and Liu et al. [20] while our work was in preparation, and have proved to be a potentially valuable model to understand this disease. DCM fibroblasts were obtained by skin biopsy of a 45 years old male, who presented with complete atrioventricular blockade associated with DCM signs, and contained a GCCA insertion at base 50 in the *lamin A/C* gene (Figure 1A and Supplementary Figure). This mutation creates a frameshift and premature stop, hence causing *lamin A/C* haploinsufficiency. The echocardiogram showed dilated right and left ventricle with left ventricular ejection fraction of 48% (*data not shown*). *Lamin A/C* truncating mutations have been described before to produce similar forms of DCM [21,22], but this mutation is novel. aWS and HGPS fibroblasts were purchased from the Coriell cell repository and contained known heterozygous mutations E578V and G608G (C1824T, creating an alternatively spliced lamin A isoform) respectively in exon 11 of *lamin A* gene (*lamin C* is not affected) (Figure 1A and Supplementary Figure). The latter 2 diseases correspond to accelerated forms of aging that affect multiple organs, but the clinical presentation is not identical [23,24]. For example, HGPS patients develop characteristic craniofacial morphology and die at young age of vascular complications (myocardial infarction and stroke), while aWS patients live longer and are phenotypically more variable. aWS is caused by mutations thought to alter the interaction of lamin A with other proteins, and in HGPS there is over-production of an abnormally processed form of lamin A, termed progerin [25], that has been proposed to interact wrongly with nuclear components as well. Lysates from fibroblasts of all 3 diseases were analyzed by Western blotting and showed reduction of lamin A/C protein in DCM, normal levels in aWS, and accumulation of progerin in HGPS compared to control fibroblasts (Figure 1B). As expected, mRNA levels of lamin A/C were comparable to the control in all cases (Figure 1C). Immunofluorescence microscopy for lamin A/C demonstrated high frequency of nuclear abnormalities in the 3 types of affected fibroblasts (Figure 1D-E). This was verified by electron microscopy, which also detected more subtle differences between them: DCM cells contained abundant nuclear pore dilatation, while aWS and HGPS displayed frequent thickening of the nuclear membrane (Figure 1F). Next, we reprogrammed the 3 types of fibroblasts into iPSCs using integrating viral vectors, either lentiviral (DCM) or retroviral (aWS and HGPS). Colonies resembling human ESCs appeared around day 20-25; they were picked around day 30 and expanded for further characterization.

### iPSCs bearing mutations in *lamin A/C* are pluripotent and display normal nuclear morphology

**Figure 2.**
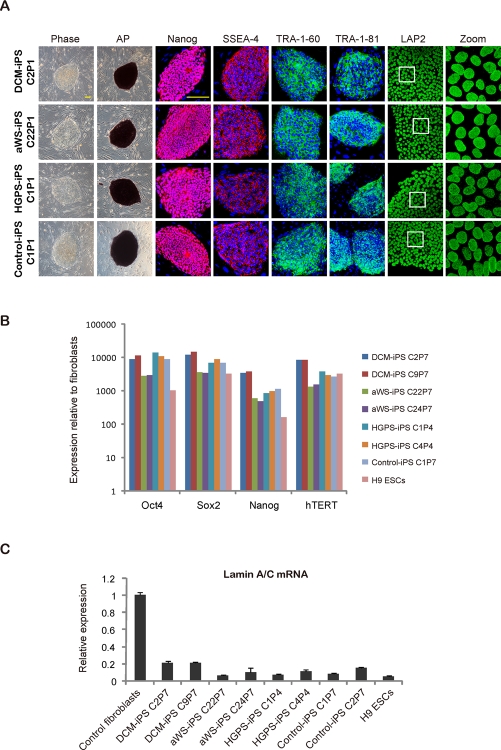
iPSCs with mutations in *lamin A/C* display human ESC-characteristics and an intact nuclear membrane **(A)** Immunofluorescence photographs for the indicated ESC markers in iPSC clones derived from DCM, aWS, and HGPS patients; iPSCs derived from normal fibroblasts were used as control. Phase contrast photographs and AP staining captures are also included. Scale bars indicate 400 μm. **(B)** qPCR for endogenous ESC transcription factors in similar iPSC clones. Values are referred to control fibroblasts; H9 ESCs and non-affectediPSCs were also added (also in **C**). **(C)** qPCR for lamin A/C mRNA in similar iPSC clones and the respective donor cells.

**Figure 3.**
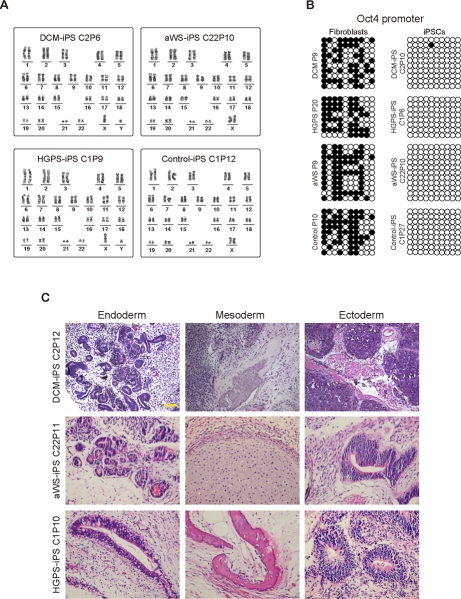
Additional characterization of iPSCs containing mutations in *lamin A/C* **(A)** Normal karyotype of representative iPSC clones from patients with the 3 diseases. **(B)** DNA methylation profile of the proximal Oct4 promoter in similar representative clones. Open circles indicate unmethylated CpGs. The respective donor cells were used as control. **(C)** Teratomas produced from representative iPSC clones from the 3 diseases contain tissues derived from the 3 germ layers.

We characterized selected iPSC colonies for all 3 diseases by standard procedures [26]. They stained positively for markers such as alkaline phosphatase (AP), SSEA-4, TRA-1-60, TRA-1-81, and the transcription factor Nanog (Figure 2A). Moreover, they displayed normal nuclear morphology as assessed by immunofluorescence for the nuclear membrane marker LAP2 (Lamina-Associated Polypeptide 2) (Figure 2A). This correlated with high expression of ESC-transcription factors (endogenous Oct4 and Sox2, plus Nanog) and hTERT (human telomerase reverse transcriptase) comparable to human ESCs and iPSCs produced from fibroblasts of a normal individual (Figure 2B). However, lamin A/C mRNA levels were much lower in all pluripotent cell lines than in control fibroblasts (Figure 2C). The latter may explain why DCM and aWS fibroblasts, and with lower efficiency also HGPS fibroblasts, could be reprogrammed in spite of their slow proliferation and increased senescence relative to the donor cells (see below Figure 5). Indeed, ESCs are known to have low expression of lamin A/C, which increases sharply upon differentiation [27]. Besides, others [19] and we (*data not shown*) have observed reduction of lamin A/C mRNA early after transduction with the exogenous factors, likely allowing the recovery of normal nuclear morphology after the initial phase of reprogramming. The diseased iPSCs also displayed normal karyotype (Figure 3A), had low levels of CpG methylation in the proximal Oct4 promoter (Figure 3B), and produced derivatives of the 3 germ layers when growth as teratomas in immunocompromised mice (Figure 3C). Therefore, we have successfully produced pluripotent cell lines by reprogramming of somatic cells from 3 independent diseases associated with mutations in *lamin A/C* mutations and they seem undistinguishable from normal ESCs/iPSCs.

### Acquisition of nuclear abnormalities upon differentiation of *lamin A/C* mutated-iPSCs

**Figure 4.**
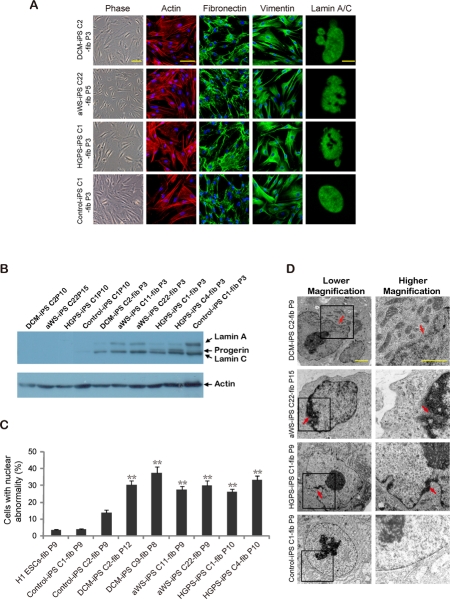
Nuclear abnormalities are restored in iPSC-derived fibroblasts containing mutations in *lamin A/C* **(A)** Immunofluorescence photographs show expression of fibronectin and vimentin, and distribution of actin in stress fibers, in iPSC-derived fibroblasts from the 3 diseases. Abnormalities of the nuclear membrane are shown on the right panels. Scale bars indicate 10 μm (lamin A/C) and 100 μm (rest). **(B)** Western blot for lamin A/C in iPSC clones from the 3 diseases and the derived secondary fibroblasts. **(C)** Quantification of cells displaying abnormal nuclear membrane by immunofluorescence microscopy. The mean of 3 independent experiments +/− SD is shown. **(D)** Electron microscopy photographs show abnormalities of the nuclear membrane in more detail for all types of secondary fibroblasts. Arrows point to more obvious defects. Scale bars indicate 1 μm.

**Figure 5.**
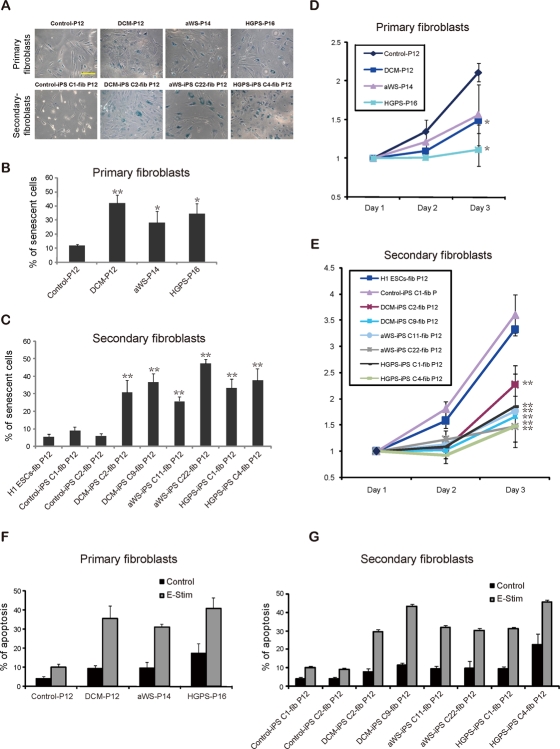
Increased senescence and susceptibility to apoptosis in iPSC-derived fibroblasts containing *lamin A/C* mutations **(A)** Phase contrast photographs after β-galactosidase staining of primary and secondary fibroblasts from the 3 diseases. Samples were measured in triplicate. Scale bars indicate 100 μm. **(B-C)** Quantification of β-galactosidase positive cells. Secondary fibroblasts from H1 ESCs were included (also in E). The mean of 3 independent experiments +/− SD is shown (also in F-G). * indicates p value <0.05. **(D-E)** Proliferation of the indicated cell types measured at different time points; a representative experiment is shown. **(F)** Electrical stimulation (E-Stim) significantly elevated the apoptotic rate in primary fibroblasts of the 3 patients compared with control fibroblasts as assessed by Annexin V apoptotic assay. **(G)** A similar trend of induced apoptosis was observed after electrical stimulation of the secondary fibroblasts from the 3 diseases.

To evaluate the potential of our iPSC cell lines for understanding the complexity of the 3 laminopathies, we differentiated them into secondary fibroblasts. The acquisition of mesenchymal-like characteristics in the iPSC-derived fibroblasts was validated by immunofluorescence microscopy for actin stress fibers, the intermediate filament vimentin and the extracellular matrix protein fibronectin. All secondary fibroblasts, including those from a non-affected iPSC clone, displayed the expected staining pattern (Figure 4A). Western blotting verified the up-regulation of lamin A/C compared to iPSCs (Figure 4B), and the acquisition of nuclear blebbing was demonstrated by immunofluorescence (Figure 4A and C) and electron microscopy (Figure 4D). Notably, the latter technique reproduced the more subtle differences in pore size and membrane thickening observed with the primary cells. Then we analyzed whether these changes were associated with cell senescence (Figure 5A-C) and slow proliferation (Figure 5D-E), as observed with the respective primary fibroblasts. Both parameters were significantly increased in secondary fibroblasts from the 3 diseases, though the tendency to display a more marked phenotype in HGPS was less obvious than in primary cells. This is probably related to the earlier onset of disease in HGPS patients and the moment in which the primary cells were collected. Likewise, we exposed primary and secondary fibroblasts to electrical stimulation because this physical stress has been shown to increase nuclear blebbing and apoptosis in vascular smooth muscle cells derived from HGPS iPSCs [19]. Primary and secondary fibroblasts displayed higher basal apoptosis compared to controls and there was synergistic increase upon electrical stimulation without significant bias among the 3 laminophaties (Figure 5F-G). Other stresses like hypoxia or gamma irradiation showed no noticeable difference between normal and diseased secondary fibroblasts (*data not shown*). In addition, we did not detect differential apoptosis after electrical stimulation between normal and affected iPSCs (*data not shown*), in agreement with their low levels of lamin A/C. Hence, reprogramming of somatic cells with mutations in *lamin A/C* reverses their abnormal phenotype thanks to reduction in protein expression, but differentiation back into the original donor cell type restores the alteration.

## DISCUSSION

We aimed to create a panel of iPSC cell lines containing distinct mutations in *lamin A/C*, as this could be used to study genotype-phenotype correlation in the respective diseases. DCM is produced by *lamin A/C* haploinsufficiency and displays a very restricted heart phenotype. As for the progerias, this term comprises several unrelated conditions in which some aspects of normal aging seem accelerated, but not all of them are laminopathies [28,29]. HGPS is termed progeria of the infancy because the clinical phenotype starts manifesting after the first years of life, while aWS clinically resembles another condition -the classical Werner syndrome- produced by mutations in the *WRN* gene [30] and causes progressive degeneration during adulthood. In both aWS and HGPS the abnormalities are systemic, though some tissues seem more susceptible. For the *in vitro* disease modeling using iPSCs we chose secondary fibroblasts because they are a clinically relevant cell type in many contexts and a well-established cell model for studying laminopathies. They reproduced the alterations of the nuclear membrane, slow proliferation, senescence, and susceptibility to apoptosis of the primary cell lines, supporting the idea that conversion to pluripotency does not affect their ability to degenerate upon differentiation. This similarity between 3 diseases that have different clinical manifestations is nevertheless puzzling. Fibroblasts likely do not cause the heart phenotype in DCM, as like in related cases the basis lies mainly in the conduction system, though we cannot formally exclude some contribution. On the other hand, DCM patients do not bear noticeable skin or osteoskeletal abnormalities, that are caused at least in part by fibroblasts, but this is a hallmark of the 2 progerias and in particular HGPS [31]. As for the appearance of heart disease in the progerias, aWS patients can actually acquire changes comparable to DCM, but HGPS patients die before these signs can develop. Therefore, it is plausible to argue that the analogous nuclear membrane and senescent/apoptotic changes observed *in vitro* for primary and secondary cells of the 3 laminopathies are a magnification caused by the tissue culture. In this regard, fibroblasts become senescent quicker outside the body context due to mitochondrial respiration and accumulation of free radical species [32]. This could imply that *in vivo* the defect of aWS, and in particular HGPS, is qualitatively similar but quantitatively stronger compared to DCM. This would explain that in DCM only those cells exposed to more substantial and continuous physical stress (like cardiomyocytes) develop the disease and with late onset, while in the progerias the defects are systemic and/or appear earlier. Even if this hypothesis were true, a different question is how functionally different mutations in *lamin A/C* can trigger the same molecular consequence. Insufficient levels of lamin A/C unavoidably cause the abnormal phenotype in DCM cells. Then, one would expect that mutant lamin A in aWS and HGPS should cause the same changes through a loss of function. But this is counterintuitive and against the current dogma, as in particular progerin is believed to act through gain of function. Besides, even though lamin A is affected in the 2 progerias, lamin C is not, and thanks to functional overlap one protein should be able to substitute the other. An alternative scenario would be that the uneven nuclear morphology and susceptibility to stress commonly used as readout for the laminopathies are to some extent misleading (at least for aWS and HGPS) and underscore different more relevant alterations. In this regard, our electron microscopy analysis has detected significant differences between primary and secondary fibroblasts for the 3 conditions. It is possible that nuclear pore changes in DCM only moderately influence trafficking in and out of this compartment but the added continuous stretching of cardiomyocytes progressively enlarges the defect and generates other abnormalities as well. On the other side, nuclear lamina thickening has been described before for HGPS primary fibroblasts and proposed to alter the anchorage of heterochromatin at the nuclear periphery [33]. This phenomenon could potentially influence the balance between activating and repressive transcripttional complexes in certain areas of the genome and thus change the epigenome [34,35]. Yet, why specific cell types (e.g. vascular smooth muscle cells and mesenchymal stem cells) seem more prone to changes *in vivo* for example in HGPS is still unclear [19]. Smooth muscle cells in the aorta are steadily exposed to stretching due to high pressure and shear stress produced by blood flow. It is thus possible that the stress in this location enhances existing epigenetic changes or combines with them. On the other hand, mesenchymal stem cells reside in parts of the anatomy where oxygen concentration is low and need to travel long distances before they can contribute to tissue repair and homeostasis [36]. Besides, upon arrival to a given tissue they undergo profound epigenetic transformations in order to acquire different cell fates, and the thickened lamina may restrict this ability. In summary, although we cannot answer yet many of these questions, our panel of cell lines and those generated elsewhere [19,20] provide a state of the art methodology to investigate them. In the future, the application of proteomic, metabolomic, or genomic approaches to a more varied differentiated progeny originated from these iPSCs may contribute to change our understanding of a group of diseases for which the existing tools were so far very limited.

## METHODS

### Ethics Statement

Investigation has been conducted in accordance with the ethical standards and according to the Declaration of Helsinki and according to national and international guidelines and has been approved by the authors' institutional review boards. Consent forms are available upon request.

### Cell culture and iPSC generation

AWS (AG04110) and HGPS (AG06917) fibroblasts were purchased from the Coriell cell repository. The mutations in all primary cells and/or the resulting iPSCs were verified by sequencing (Supplementary Figure). All fibroblasts were maintained in DMEM (Invitrogen) + 10% fetal bovine serum (FBS) (Hyclone). Fibroblasts at passage 2 (DCM), 9 (aWS) and 17 (HGPS) were transduced with a cocktail of retroviruses (producing Sox2, Klf4, Oct4 and c-Myc) or lentiviruses (Sox2, Klf4, Oct4, c-Myc, Lin28 and Nanog) as reported before [26,37,38]. Fibroblasts from a normal individual were reprogrammed with the above lentiviruses and the primary cells and/or iPSCs were used as controls where indicated. Picked iPSCs and human ESCs were routinely maintained on feeders (mitotically inactivated-murine embryonic fibroblasts) in human ESC medium (DMEM/F12 plus 20% KnockOut serum replacement (KSR, Invitrogen), penicillin/streptomycin, 1 mM Glutamine, 0.1 mM non-essential amino acids, 0.1 mM beta-mercaptoethanol and 8 ng/ml bFGF (Shenzhen Symmix Industry) or on Matrigel (BD Biosciences) in mTeSR1 (Stemcell Technologies).

### iPSC characterization

AP staining, transgene integration, karyotyping and bisulfate sequencing were performed following standard procedure. For aWS and HGPS we also performed single tandem repeat analysis (Kingmed) to show a match between donor cells and iPSCs (*data not shown*). Genomic DNA was extracted using the DNeasy Tissue kit (Qiagen) and total RNA was extracted using Trizol (Invitrogen). qPCR was performed using a Thermal Cycler DiceTM Real Time System (ABI7300, ABI) and SYBR Green Premix EX TaqTM (Takara); beta actin or GAPDH were used for normalization and all items were measured in triplicate (SD is included in some graphs). qPCR primers used in this study are available upon request. Secondary fibroblast differentiation was done following a reported protocol [39]. For teratomas, iPSCs were injected subcutaneously or intramuscularly into the right hind leg of immuno-compromised NOD-SCID mice. Tumors were excised after 8 weeks, fixed, embedded in paraffin, sectioned and stained with hematoxylin/eosin.

### Immunofluorescence microscopy, electron microscopy and Western blotting

For immunofluorecence, cells were fixed in 4% paraformaldehyde for 30 minutes, washed, blocked and permeabilized in blocking solution (PBS containing 3% normal goat serum and 0.2% Triton X-100) for 30 minutes. Then they were incubated with primary antibodies in blocking solution at 4°C overnight, washed twice and incubated with the corresponding secondary antibodies for 1 hour at room temperature. Cells were washed twice and stained with DAPI (Sigma) for 5 minutes, and then observed and photographed using a LEICA DMI6000B microscope (Leica Microsystems GmbH). For quantification of nuclear abnormalities ten fields were counted per slide and 3 slides were counted per experiment. For electron microscopy primary and secondary fibroblasts were harvested and washed in PBS. The cell pellets were fixed in 2% paraformaldehyde and 1% glutaraldehyde in 0.1 M sodium cacodylate-HCl buffer pH 7.4. Next, they were then stained with 1% osmium tetroxide (OsO_4_) in cacodylate buffer for 1 hour at room temperature and washed twice in 0.1 M sodium cacodylate-HCl buffer. The cells were then embedded in 2% agarose, dehydrated and embedded in epoxy resin. Ultrathin sections were examined by transmission electron microscopy using a Philips EM208s microscope. Western blot membranes were developed using Immobilon Western Chemiluminescent HRP Substrate (Millipore). Primary antibodies were purchased from: lamin A/C Santa Cruz, LAP2 BD Biosciences, fibronectin and vimentin Sigma, Nanog R&D, SSEA-4 Abcam, TRA-1-60 and TRA-1-81 Millipore.

### β-galactosidase/Annexin V assays, proliferation and electrical stimulation

β-galactosidase detection was performed using a kit (Cell Signalling Technology Inc) according to the manufacturer's instructions. Senescent cells were indicated by the development of blue color under the microscope at 200×. Blue cells in 20 random fields were counted in duplicate wells of each experiment. Annexin V apoptotic assay was performed using a kit according to manufacturer's instruction (BD Biosciences). The percentage of apoptotic cells was measured by flow cytometry (Beckman Coulter) and 10,000 events were counted. Proliferation was measured using the MTT assay, samples were measured in triplicate. For electrical stimulation primary and secondary fibroblasts were seeded at 1 × 10^5^ cells per well (in triplicate) on 6-wells plates (Nunc A/S) and subjected to electric field stimulation with an eight-channel C-Pace chronic stimulator (Ion-Optics Co., MA) at 75V/cm, 1 Hz, 2ms for 24 hours.

## SUPPLEMENTARY MATERIALS


