# Supplementary figures and images for: Generation of induced pluripotent stem cell lines from 3 distinct laminopathies bearing heterogeneous mutations in lamin A/C
Source: Aging (Albany NY). 2011 Mar 28;3(4):380–90. doi: 10.18632/aging.100277 (PMC3117453; doi:10.18632/aging.100277)

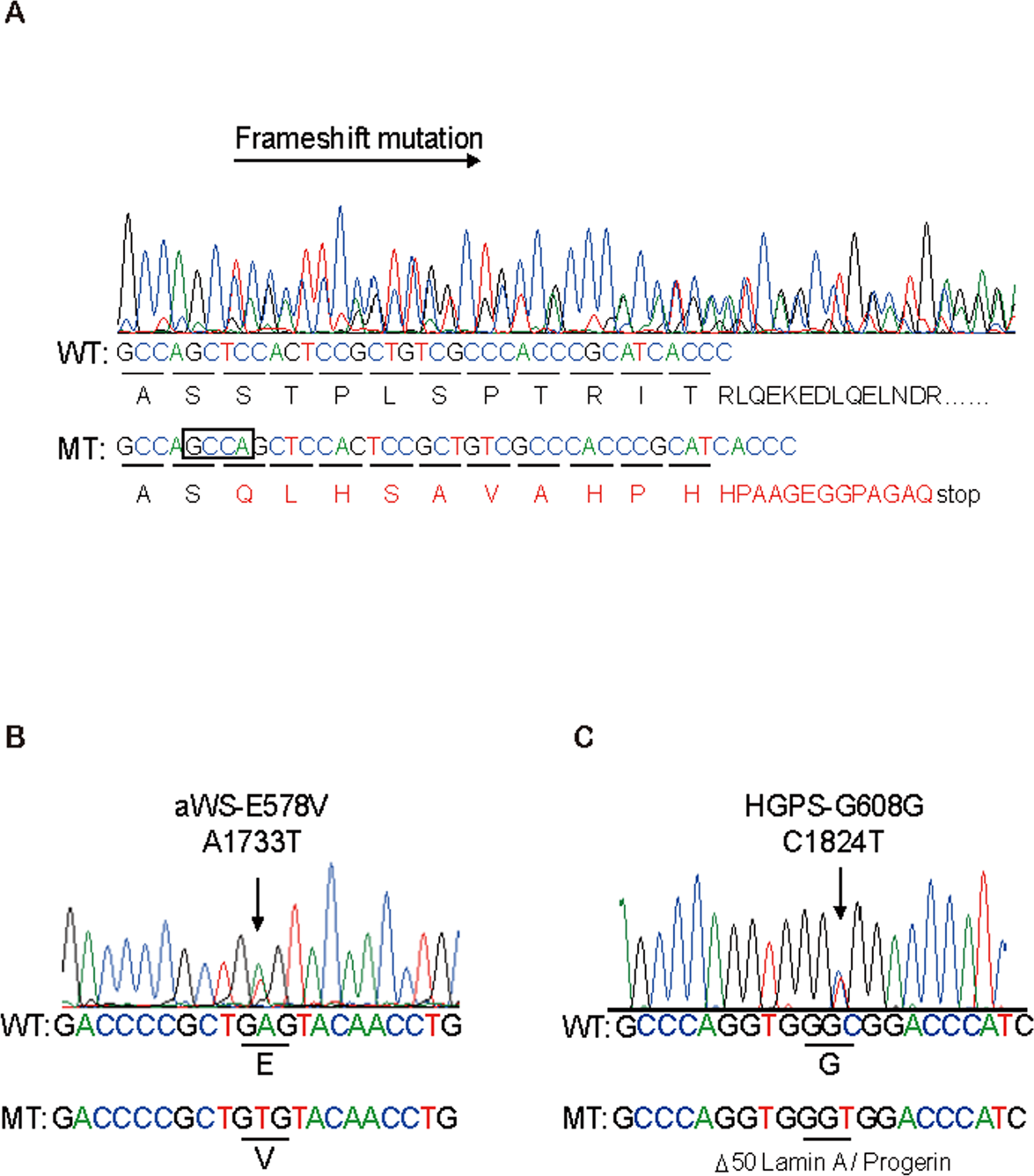

Supplement: Supplementary file 1 [file aging-03-380-s001.tif]
